# Selectively Adsorptive Extraction of Phenylarsonic Acids in Chicken Tissue by Carboxymethyl α-Cyclodextrin Immobilized Fe_3_O_4_ Magnetic Nanoparticles Followed Ultra Performance Liquid Chromatography Coupled Tandem Mass Spectrometry Detection

**DOI:** 10.1371/journal.pone.0107147

**Published:** 2014-09-12

**Authors:** Jing Jia, Wei Zhang, Jing Wang, Peilong Wang, Ruohua Zhu

**Affiliations:** 1 Key Laboratory of Agrifood Safety and Quality, Ministry of Agriculture, Beijing, P.R. China; 2 Department of Chemistry, Capital Normal University, Beijing, China; 3 Institute of Quality Standards and Testing Technology for Agriculture Products, China Agricultural Academy of Science, Beijing, P.R. China; Brandeis University, United States of America

## Abstract

Carboxymethyl α-cyclodextrin immobilized Fe_3_O_4_ magnetic nanoparticles (CM-α-CD-Fe_3_O_4_) were synthesized for the selectively adsorptive extraction of five phenylarsonic acids including *p*-amino phenylarsonic acid, *p*-nitro phenylarsonic acid, *p*-hydroxy phenylarsonic acid, *p*-acylamino phenylarsonic acid and *p*-hydroxy-3-nitro phenylarsonic acid in chicken tissue. Using ultra performance liquid chromatography coupled with tandem mass spectrometry (UPLC-MS/MS), a highly sensitive analytical method was proposed for the determination of five phenylarsonic acids. It was shown that CM-α-CD-Fe_3_O_4_ could extract the five phenylarsonic acids in complex chicken tissue samples with high extraction efficiency. Under the optimal conditions, a high enrichment factor, ranging from 349 to 606 fold, was obtained. The limits of detection (LODs) (at a signal-to-noise ratio of 3) were in the range of 0.05–0.11 µg/kg for the five phenylarsonic acids. The proposed method was applied for the determination of five target phenylarsonic acids in chicken muscle and liver samples. Recoveries for the spiked samples with 0.2 µg/kg, 2.0 µg/kg and 20 µg/kg of each phenylarsonic acids were in the range of 77.2%–110.2%, with a relative standard deviation (RSD) of less than 12.5%.

## Introduction

**Figure 1:**
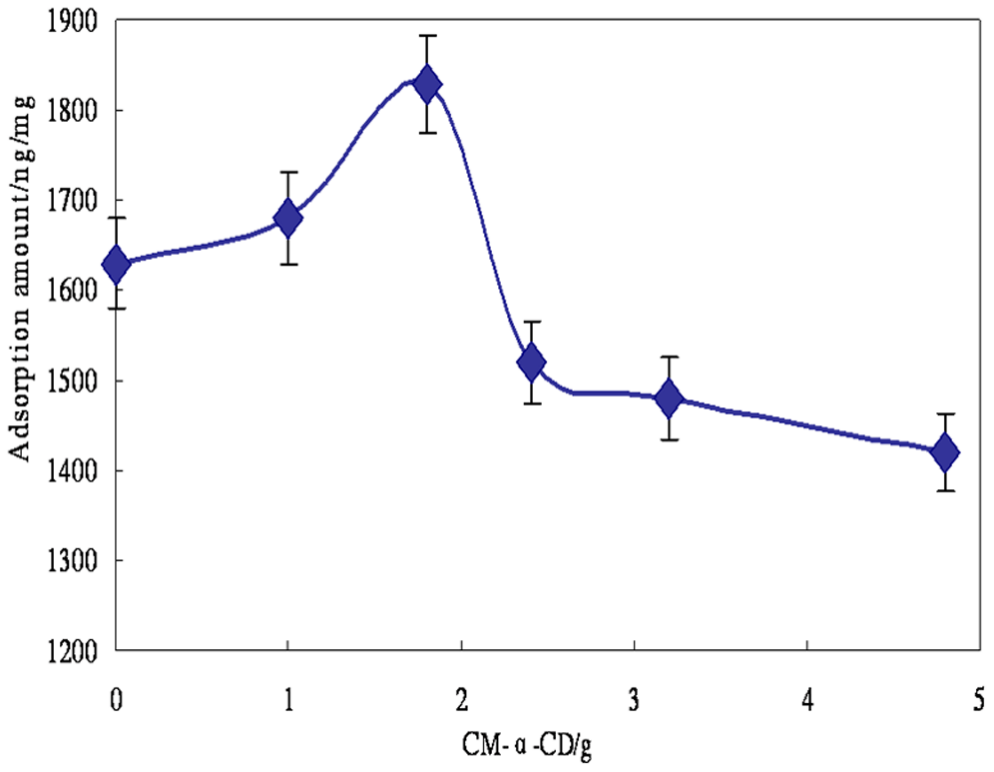
Effect of CM-α-CD amount used in modification on the adsorption efficiency of phenylarsonic acids (ROX). (n = 3, with RSD<6.5%).

4-Hydroxy-3-nitrobenzenearsonic acid, also known as roxarsone (ROX), has been used since 1944 as a feed additive in the poultry industry to promote growth and to control coccidiosis, a parasitic disease that infects the intestinal tract of poultry [1]. Besides ROX, some other organic arsenic compounds, including p-amino phenylarsonic acid (p-APAA), p-nitro phenylarsonic acid (p-NPAA), p-hydroxy phenylarsonic acid (p-HPAA) and p-acylamino phenylarsonic acid (AAPAA) (**Fig. 1**) have been successively employed for the same purposes. Their slight structural difference, i. e. different substituent groups on the aromatic ring, results in different growth-promoting and disease-controlling effects [2]. Phenylarsonic acids have been approved as feed additives by many countries at levels of 25–50 mg/kg [3]. Recent studies showed that phenylarsonic acids in the environment might be converted into elemental arsenic and other inorganic arsenic compounds, which are known to be strongly carcinogenic [4]. Some countries in EU strictly control the use of phenylarsonic acid additives, while in the U.S., Tyson Foods, the country's largest poultry producer, stopped the use of arsenic compounds in 2004. After the release of 2011 FDA report of elevated inorganic arsenic in the livers of chickens treated with ROX, Pfizer Animal Health, the US manufacturer of ROX, quickly suspended ROX sales [5]. In order to monitor the residues of phenylarsonic acid in animal products, it is of great significance to establish a convenient, sensitive and reliable method to analyze the organic arsenic in samples [6].

Several analytical methods for the determination of phenylarsonic acids in the environment have been reported, including liquid chromatography (LC) coupled to atomic absorption spectroscopy (AAS) [7], atomic emission spectroscopy (AES) [8] or atomic fluorescence spectrometry (AFS) [9] as well as gas chromatography-mass spectrometry (GC-MS) [10], capillary electrophoresis (CE) coupled to ultraviolet and visible light detector [11] or inductively coupled plasma-mass spectrometry (ICP-MS) [12]. LC has been demonstrated to be the most effective method in arsenic separation, and ICP-MS can provide low detection limit. Therefore, LC-ICP-MS is one of the most powerful research means for analysis of organic arsenic in complex samples [2], [13]. The LC-MS/MS is a powerful separation and detection platform in multi residues analysis. Pergantis et al [14] have developed a stable method for determination of 5 phenylarsonic acids including ROX, p-APAA, p-NPAA, AAPAA, p-HPAA and other organic arenics using LC-MS/MS in positive ionization mode. The LODs of the developed method achieved sub ng/g level, whereas the analytical time was too long. Furthermore, compared with other arsenic speciation methods, the LC-MS/MS could provide more structural information of the phenylarsonic acids.

After feeding, nearly all phenylarsonic acids are excreted unchanged to the environment through the disposal of poultry litter, so the residues in animal tissues are very low [15], [16]. Some studies indicated that the approved conditions of use mandate a 5-day withdrawal period from the medicated feed before animals are slaughtered, and limits are in place for total residues of combined arsenic (As) in meat from ROX treated animals [0.5 mg kg^−1^ As in muscle tissue and eggs, and 2 mg kg^−1^ As in liver and kidney] [17]. To further lower the detection limits of phenylarsonic acids in complex biological samples, nano-materials have been used to selectively extract and concentrate arsenic compounds. The most commonly used nano-materials for the adsorption of arsenic compounds include goethite [18], titania [19], iron oxide [20], [21], Fe_3_O_4_ nanoparticles [22], [23] or modified Fe_3_O_4_ nanoparticles [24]. Among these nano-materials, Fe_3_O_4_ nanoparticles are well suited for arsenic analysis due to the following advantages. First, they can be easily isolated from solutions by applying an external magnetic field [25], which ensures simplified sample adsorption and elution processes. Second, Fe_3_O_4_ nanoparticles have been demonstrated to have higher affinity toward arsenic element than other nano-materials, resulting in higher arsenic extraction efficiency. Fe_3_O_4_ magnetic nanoparticles have been used for removing inorganic arsenic ions in water sample. For example, Fe_3_O_4_ nanoparticles dispersed in chelating resins or coated with adequate chelating agents have been used for the removal of a wide range of metal ions from wastewater, overall displaying higher adsorption capacity than traditional materials [26]–[29]. More recently, β-CD coated Fe_3_O_4_ nanoparticles have been successfully applied for the removal of methylene blue and copper ions [30], [31]. It is found that the cavity of cyclodextrin and its surface hydroxyl group can impart better binding capability and chemical stability to the magnetic particle [32].

In this work, a highly sensitive determination method was established to monitor five phenylarsonic acids in chicken tissues. In our first attempt, it was found that the adsorption of phenylarsonic acids by Fe_3_O_4_ magnetic nanoparticles was not as good as inorganic arsenic compounds. It was then decided to use CM-α-CD to couple on the surface of Fe_3_O_4_ providing CD cavities to fit the benzene rings in the structures of phenylarsonic acid compounds. In addition, hydrogen bonding and electrostatic interactions between hydroxyl/carboxyl groups of modified CM-α-CD and amino/nitro of phenyl arsenic acids were also increased. The adsorption properties of modified Fe_3_O_4_ magnetic nanoparticles to phenylarsonic acids were studied, and the interactions between nanoparticles and phenylarsonic acids were examined. The synthesized materials were successfully applied in the sample clean-up and pre-concentration of phenylarsonic acids in chicken muscle and liver samples, which were subsequently separated and detected by UPLC-MS/MS.

## Materials and Methods

### Apparatus

ICP-MS (Agilent7500Ce, USA) was used to study the adsorption properties of synthesized material for phenylarsonic acids. The optimum operation parameters of ICP-MS were selected by tuning. The power was 1550W, the flow rates of cooling air, auxiliary air and carrier were 15.0 L/min, 1.0 L/min and 1.12 L/min, respectively. The sample rate of ascension by using peristaltic pump was set as 1.0 mL/min. The integration time for arsenic was 0.3 s/isotope. The operating parameters of UPLC-MS/MS (Waters Xevo TQ, USA) were as follows: capillary voltage = 2.8 kv, desolvation temperature = 450°C, desolvation gas flow rate = 600 L/Hr. The mobile phase was a mixture of acetonitrile (solvent A) and water containing 0.1% formic acid (solvent B) at a flow rate of 0.3 mL/min. All chromatographic separations were carried out in linear gradient mode as follows. In the first minute, solvent A was maintained at 98% (v/v). Solvent B quickly dropped to 30% from 1 to 3 min, followed by dramatic increase back to 98% from 3 to 5 min. MS parameters of UPLC-MS/MS are showed in **Table 1**.

**Table 1:** Analytical parameters of MS/MS.

Compounds	Molecular weight	Precursor Ion (m/z)	Product Ion (m/z)	Cone voltage (V)	Collision voltage (V)
p-APAA	217	218	92	27	21
			109	27	16
p-HPAA	218	219	110	28	18
			201	28	13
p-NPAA	247	248	202	30	18
			230	30	14
AAPAA	260	261	244	29	14
ROX	263	264	246	33	14

The closed microwave digestion system (CEM MARS, American) was used to digest samples for the determination of the total arsenic in the crude samples. The homogenizer (IKA, Germany) was applied to sample pretreatment and rocking hammock bed was from Zhicheng, Shanghai, China.

### Standard solutions and reagents

Five phenylarsonic acids (98%) were obtained from the Chinese Academy of Agricultural Sciences. The standard stock solutions were prepared by dissolving each arsenic species in pure water at an arsenic concentration equivalent to that of 1 mg/mL phenylarsonic acids and stored at 4°C in the dark.

Reagents for preparing magnetic nanoparticles: FeCl_2_⋅4H_2_O and FeCl_3_⋅6H_2_O (analytical reagent grade) were purchased from Tianjin Guangfu Fine Chemical Research Institute. Sodium chloroacetate (98%) was bought from Alfa Aesar. α-cyclodextrin (98%) was purchased from Beijing Dilang Biochemical Technology Co., Ltd., China. Other reagents including methanol, ethanol, acetone, toluene, formic acid and sodium hydroxide were of analytical reagent grade and all bought from Beijing Chemical Plant. The water used throughout the experiment was purified using a Milli-Q water purification system (Millipore, Germany).

### Preparation of CM-α-CD stabilized magnetic nanoparticles

#### Synthesis of CM-α-CD

CM-α-CD was prepared according to the following procedures. α-CD (3.55 mmoL) and NaOH (90.2 mmoL) were first dissolved into 20 ml water. The solution was then heated at 90°C for 5 min, followed by the addition of 74.6 mmoL sodium chloroacetate. The solution was heated for 3 h at 90°C under stirring. Once cooled to room temperature, the pH of the solution was adjusted to 6–7 by hydrochloric acid. The nearly neutral solution obtained was then poured into about 500 mL methanol. CM-α-CD was precipitated out as white solids, which was filtered and washed with methanol for a few times and then dried under vacuum for 3 d at 50°C and 0.085 MPa. The melting point of the CM-α-CD product was about 245.5°C as determined by micro melting point apparatus.

#### Synthesis of Fe_3_O_4_ nanoparticles

Fe_3_O_4_ magnetic nanoparticles were prepared according to the conventional co-precipitation method [33]. A mixture of 2.0 g FeCl_2_⋅4H_2_O and 5.2 g FeCl_3_⋅6H_2_O was added into a 500 mL conical flask containing 200 ml 0.05 M HCl. After dissolution of the solids, 250 ml 0.75 M NaOH solution was poured into the flask under a blanket of N_2_. The mixture was stirred for another 2 h at 80°C. The Fe_3_O_4_ nanoparticles were then obtained in the form of black precipitates, which were separated with a magnet and washed subsequently by water (3 times) and ethanol (twice). It should be noted that both the HCl and NaOH solutions were degassed by a sonicator for 20 min before use.

#### CM-α-CD modified Fe_3_O_4_ nanoparticles (CM-α-CD-Fe_3_O_4_)

The Fe_3_O_4_ nanoparticles prepared in the previous step were added into 60 ml PBS buffer solution (pH = 6.6) containing 1.6 g of CM-α-CD. The suspension was sonicated for 3 min and then stirred for 3 h at 80°C. After cooling to room temperature, the nanoparticles were washed several times by PBS buffer solution to remove excess CM-α-CD. The CM-α-CD modified Fe_3_O_4_ nanoparticles were then dried at 80°C in a vacuum oven.

### Adsorption procedure

#### Static adsorption

In a 10 mL centrifuge tube, 5 mg CM-α-CD-Fe_3_O_4_ nanoparticles were mixed with 8 mL standard solution of phenylarsenic acid with a given concentration. The centrifuge tube was placed on a rocking hammock bed at a rate of 270 rpm. After equilibrating for 30 min, the magnetic nanoparticles were separated from the solution with external magnetic field. The nanoparticles were rinsed twice with ethanol and dried in N_2_. To desorb target compounds, 1.0 mL pure water was added to the nanoparticles followed by equilibration for 10 min. The aqueous solution containing target compounds was filtered through a 0.22 µm Poly (ether sulfones) (PES) syringe filter and analyzed by ICP-MS. Standard phenylarsenic acid solutions of other concentrations were analyzed in the same way.

#### Dynamic adsorption

In a 10 mL centrifuge tube, 5 mg CM-α-CD-Fe_3_O_4_ nanoparticles were mixed with 8 mL standard solution of phenylarsenic acid with a given concentration. A number of centrifuge tubes were prepared in this manner for a given concentration of standard. The centrifuge tubes were placed on an orbital shaker at a rate of 270 r/min. At different time points, one tube was removed and the magnetic nanoparticles contained in the tube were separated from the solution with external magnetic field. The following washing, desorption and ICP-MS analysis procedures were the same as those in the static adsorption step. Standard phenylarsenic acid solutions of other concentrations were analyzed in the same way.

### Sample analysis

Chicken tissues including meat and liver were bought from a supermarket in Beijing. Chicken meat and chicken liver samples were pulverized and freeze-dried for 24 h. The freeze-dried sample was homogenized by grinding and frozen until analysis. In a 50 mL centrifuge tube, 5.0 g freeze-dried chicken tissue sample and 10 mL ethanol were added. The extraction was repeated twice, each lasting 30 min. The combined extracts were equilibrated with 5 mg CM-α-CD-Fe_3_O_4_ nanoparticles for 10 min. Then the magnetic nanoparticles which adsorbed target analytes were separated under external magnetic field, the analytes adsorbed on the nanoparticles were then desorbed with 2 mL deionized water. The aqueous solution containing target compounds was filtered through a 0.22 µm PES syringe filter and analyzed by UPLC-MS-MS.

### Determination of total arsenic

The total amount of arsenic in chicken tissue samples was determined by microwave digestion ICP-MS according to reference [34]. Each digestion can containing 0.5 g chicken tissues samples was added 5 mL 65% nitric acid. Stages digestion method by controlling temperature was used. The obtained digestion solution was diluted until the concentration of nitric acid fell below 5% and then subjected to ICP-MS analysis.

## Results and Discussion

### Optimization of adsorption efficiency

**Figure 2:**
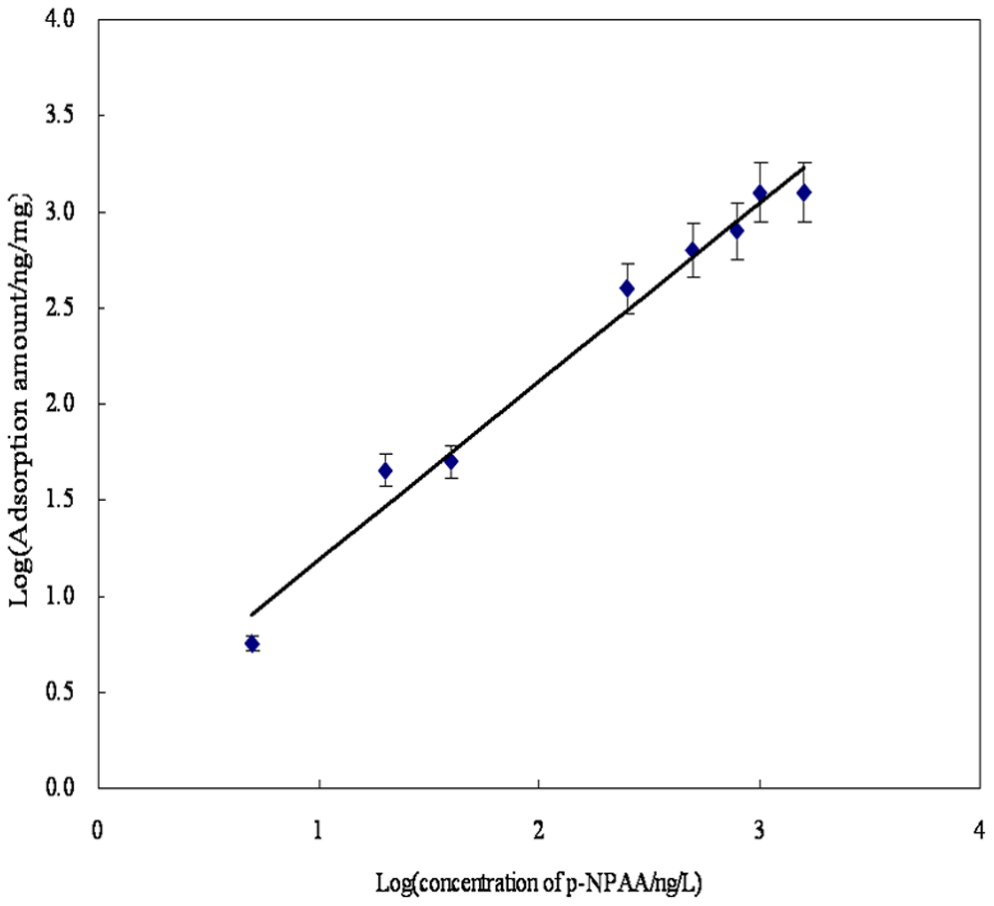
Recovery of different concentrations of phenylarsonic acids (ROX). (n = 3, with RSD<5.0%).

**Figure 3:**
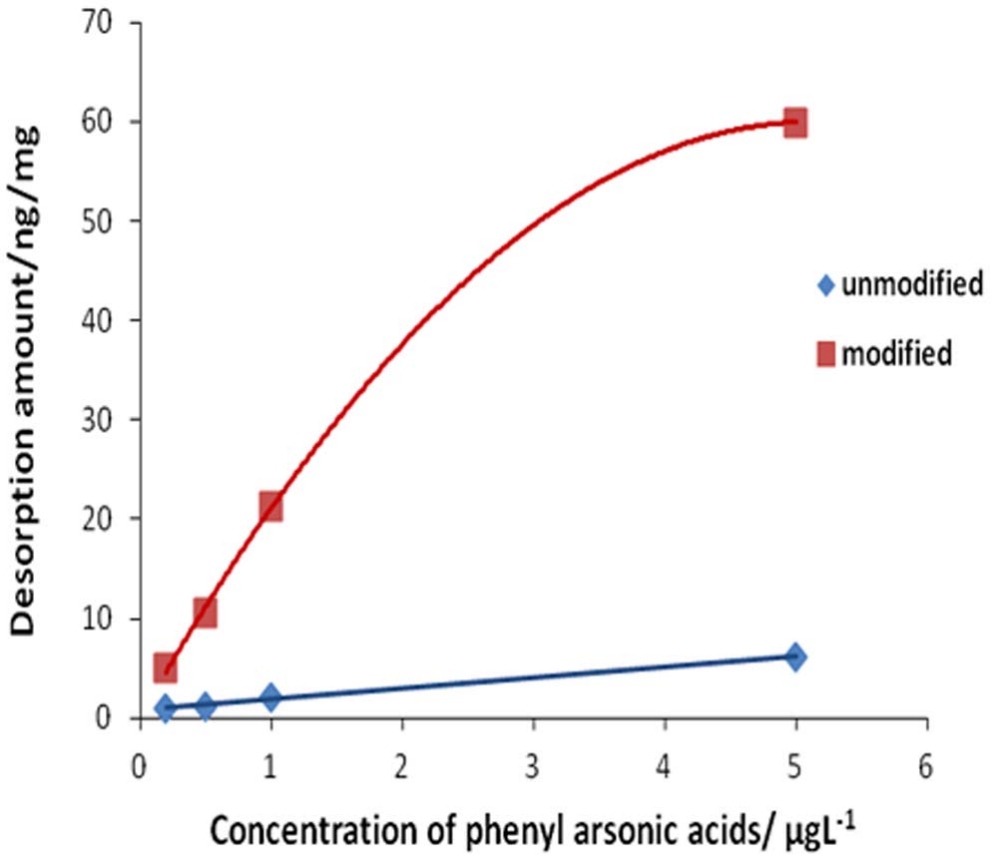
Comparison of Fe3O4 nanoparticles (blue diamond) and CM-a-CD-Fe3O4 nanoparticles for adsorption of ROX (red square).

For optimal adsorption efficiency, the amounts of CM-α-CD for the modification of Fe_3_O_4_ nanoparticles were varied in six dosages of 0.8, 1.6, 2.4, 3.2, and 4.8 g to obtain 450 mL of Fe_3_O_4_ suspension as described in section 2.3.2. The adsorption was carried out by equilibrating 5 mg CM-α-CD-Fe_3_O_4_ in 8 mL ROX standard solution at a concentration of 1 µg/L. The adsorption efficiency was then determined by ICP-MS. As shown in **Fig. 1**, the highest adsorption efficiency corresponds to 1.6 g CM-α-CD. **Fig. 2** shows the adsorption behaviors of ROX at different concentrations by CM-α-CD-Fe_3_O_4._ The amount of adsorbed ROX is linear over a large concentration range of ROX. To verify the important role of CM-α-CD in the adsorption of phenylarsonic acid, the adsorption efficiencies of modified and unmodified Fe_3_O_4_ nanoparticles for p-NPAA were compared. As shown in **Fig. 3**, modified material was obviously superior to unmodified material.

### Characterization of magnetic CM-α-CD-Fe_3_O_4_

**Figure 4:**
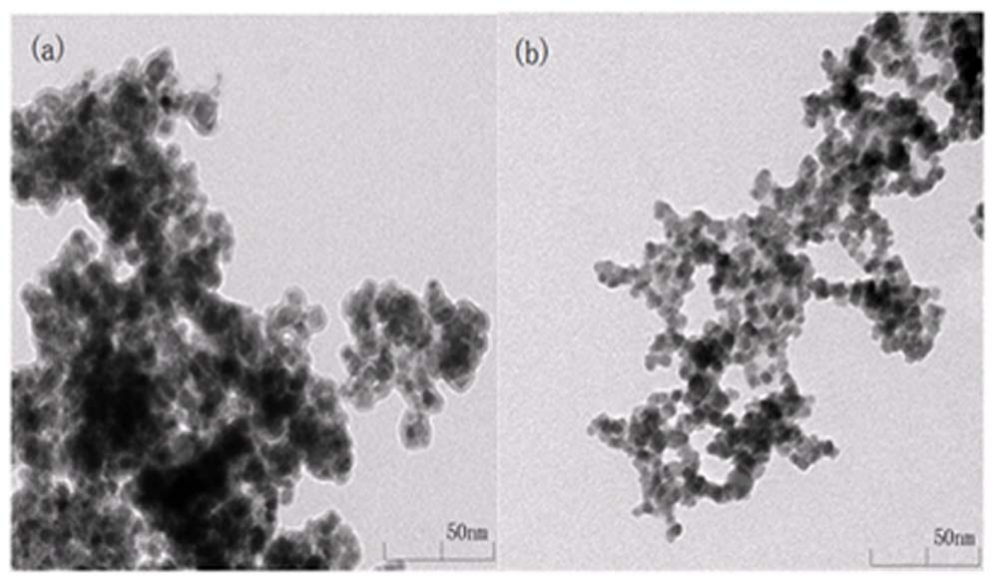
TEM images of magnetic Fe_3_O_4_ nanoparticles (a) and CM-α-CD-Fe_3_O_4_ nanoparticles (b).

The transmission electron microscope (TEM), fourier transform infrared spectrometry (FTIR) and thermo gravimetric analyzer (TGA) were used to characterized the magnetic nanoparticles. The TEM images of Fe_3_O_4_ and CM-α-CD-Fe_3_O_4_ nanoparticles are shown in **Fig. 4** **(a)** and **Fig. 4** **(b)**, respectively. Unmodified Fe_3_O_4_ nanoparticles, approximately 10 nm in diameter, trend to aggregate because of size effect of Fe_3_O_4_ nanoparticles. On the other hand, CM-α-CD-Fe_3_O_4_ nanoparticles shown in **Fig. 4** **(b)** are much better dispersed in an aqueous solution with diameters of about 5 nm, because the Fe_3_O_4_ nanoparticles were modified with CM-α-CD and the surface of Fe_3_O_4_ nanoparticles was protected by CM-α-CD. It can be observed in **Fig. 4** **(b)** that the composite of modified nanoparticles was more compacted and displayed roughly spherical shapes.

The FTIR spectrums of Fe_3_O_4_, CM-α-CD and CM-α-CD- Fe_3_O_4_ nanoparticles respectively were scanned (See Fig. S1). In all three samples, a strong characteristic O-H absorption band at around 3400 cm^−1^ is clearly visible. There is a strong Fe-O absorption peak at 580 cm^−1^ for both Fe_3_O_4_ and CM-α-CD-Fe_3_O_4,_ suggesting the intactness of the Fe-O bond during the modification process. Compared with the innate Fe_3_O_4_ nanoparticles, new characteristic peaks at 1100 and 2900 cm^−1^ appeared in the spectrum of CM-α-CD- Fe_3_O_4_ nanoparticles, corresponding to C-O-C and C-H groups of CM-α-CD, which are also visible in the spectrum of pure CM-α-CD. This confirms the success of the modification of CM-α-CD to Fe_3_O_4_ nanoparticles.

The amount of CM-α-CD grafted on the surface of Fe_3_O_4_ and the number of CM-α-CD molecules immobilized on a single Fe_3_O_4_ nanoparticle can be estimated from the TGA results. It is known that CM-α-CD decomposes completely above 600°C.The TGA curves of Fe_3_O_4_ and CM- α-CD-Fe_3_O_4_ exhibit two steps of weight loss. In both cases, the first step can be attributed to the loss of residual water.The amounts of CM-α-CD coated on the surface of Fe_3_O_4,_ calculated on the basis of the second-step loss, are 5.3% and 9.6% for Fe_3_O_4_ nanoparticles and CM-α-CD-Fe_3_O_4_ nanoparticles, respectively. The increase of the weight loss can be ascribed to CM-α-CD grafted on Fe_3_O_4_ nanoparticles (about 43 mg/g). According to Y. P. Wu [35], the number of CM-α-CD molecules immobilized on a single Fe_3_O_4_ can be calculated in equation (1): 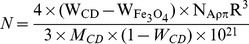
(1)where N is the number of CM-α-CD molecules immobilized on each Fe_3_O_4_, R is the mean radius of CM-α-CD-Fe_3_O_4_ (5.0 nm based on the TEM results), ρ is the density of the nanoparticle (4.8 g/cm^3^), N_A_ is Avogadro's number, W_CD_ and W_Fe3O4_ are the weight losses of the CM-α-CD-Fe_3_O_4_ and Fe_3_O_4_ respectively, M_CD_ is the molar mass of CM-α-CD immobilized on CM-α-CD-Fe_3_O_4_. The calculated number of CM-α-CD molecules immobilized on each CM-α-CD-Fe_3_O_4_ is about 70.

### Selection of adsorption solvent

**Figure 5:**
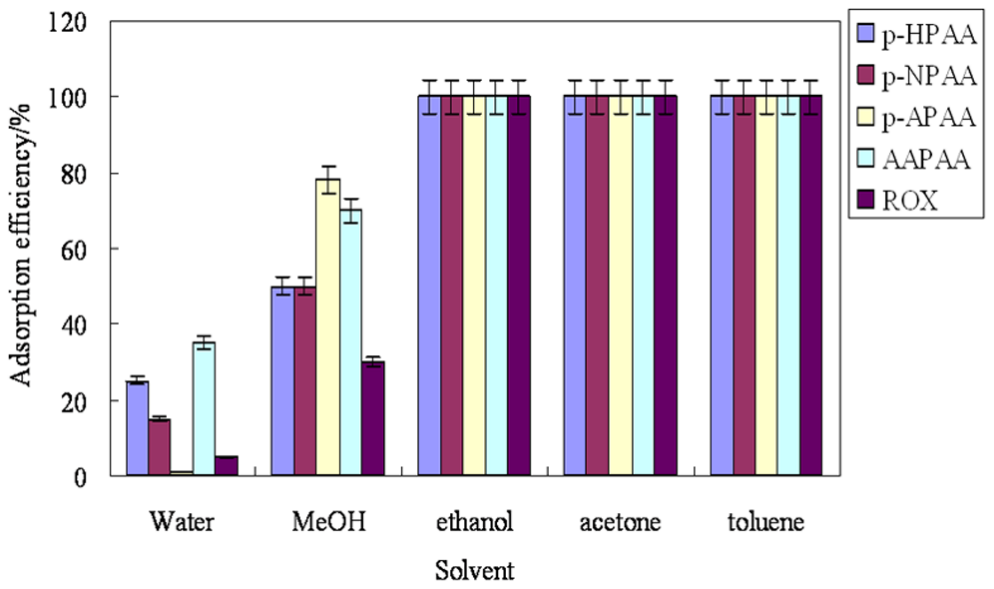
Effect of adsorption solvent on the adsorption efficiency of five phenylarsonic acids. The concentration of each phenylarsonic acids was 50 ng/mL. Adsorption time was 30 min. 5 mg adsorbent was used. (n = 3, with RSD<4.5%).

The solvent for phenylarsonic acid solutions is an important factor affecting the adsorption efficiency. Five candidate solvents, i.e., water, methanol, ethanol, acetone and toluene, were compared (Fig. 5). The adsorption efficiency of all target arsenic species could reach 100% in ethanol, acetone and toluene. Ethanol was selected as the solvent in the following experiments due to the relatively low toxicity. In water and methanol, the adsorption efficiencies of the five target compounds are very poor, which might be because of the water and methanol possess higher polarity than ethanol, acetone and toluene.

### Static adsorption

#### Saturation of adsorption

**Figure 6:**
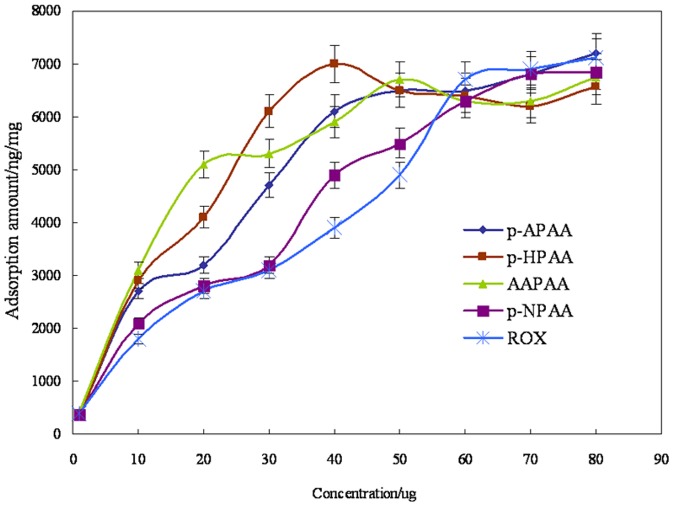
Adsorption saturation curve of CM-α-CD-Fe_3_O_4_ nanoparticles to five phenylarsonic acids. 5 mg adsorbent was used for each solution. Adsorption time was 30 min. Desorption was in water for 10 min. (n = 3, with RSD<5.7%).

The adsorption saturation curve is shown in **Fig. 6**. The point of saturation was reached at 40 µg for p-HPAA, p-APAA, and AAPAA, whereas it was 60 µg for p-NPAA and ROX, corresponding to about 0.2 µmol of each phenylarsonic acid.

The amount of CM-α-CD coated on the surface of Fe_3_O_4_ could be estimated from the saturation of adsorption. Since one phenylarsonic acid molecule was supposed to fit one CD cavity, there should be approximately equal amount of CD and phenylarsonic acid. Thus, the amount of CM-α-CD might be calculated through its molecular mass of 1029 according to equation (2). The estimated amount of grafted CM-α-CD was about 41.2 mg/g, which was similar to 43 mg/g calculated by TGA in 3.2.3. 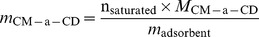
(2)


#### Enrichment factors of low concentration

The lowest concentrations of phenylarsonic acids that could be absorbed by CM-α-CD-Fe_3_O_4_ as well as enrichment factors were investigated. Ultimately, the enrichment factors of p-APAA, AAPAA, ROX, p-HPAA and p-NPAA were 414, 426, 581, 606 and 349 (theoretical enrichment factor was 500) at the concentration of 0.1 µg/L. Enrichment efficiency was 69.8%-121.2% and experimental results are shown in **Table 2**.

**Table 2:** Enrichment factors of CM-α-CD-Fe_3_O_4_ for 0.1 µg/L of each phenylarsonic acids in toluene solvent.

Phenylarsonic acids	Theory concentration (µg/L)	Experimental concentration (µg/L)	Enrichment factor	Enrichment Efficiency (%)
p-APAA	50.0	41.4	414	82.8
AAPAA	50.0	42.6	426	85.2
ROX	50.0	58.1	581	116.2
p-HPAA	50.0	60.6	606	121.2
p-NPAA	50.0	34.9	349	69.8

### Dynamic adsorption

#### Optimization of adsorption time and desorption time

The water was selected as desorption solvent. The adsorption efficiency was optimized by varying the adsorption time in the range of 1–40 min with all other parameters held constant. The adsorption efficiency increased with the adsorption time from 1 to 30 min followed by a plateau. The rate of adsorption was so high that 5 min was enough to adsorb the target compounds. Similarly, desorption efficiency was optimized by varying desorption time in the range of 1–10 min. The desorption efficiency increased with desorption time till 10 min, at which point it plateaued. Therefore, 10 min was selected as desorption time and desorption efficiency was achieved above 75%.

#### The tolerance of coexisting inorganic ions and organic analogues

**Figure 7:**
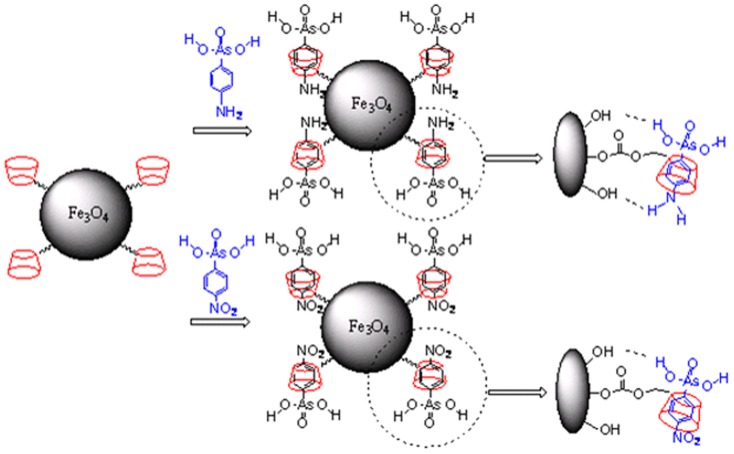
The likely interactions between CM-α-CD-Fe_3_O_4_ and the acids p-APAA and p-NPAA.

By fixing the concentration of each phenylarsonic acids at 50 µg/L and 12 coexisting inorganic ions including Mn^2+^, Cu^2+^, K^+^, Zn^2+^, Ba^2+^, Fe^3+^, Mg^2+^, Ca^2+^, Pb^2+^, Cr^3+^, Cd^2+^, and Sn^2+^ at concentrations of 500, 5000, and 50000 µg/L for each inorganic ions respectively, interference of inorganic ions on the adsorption of phenylarsonic acids was studied. No significant interferences were observed under the optimum conditions described above in the presence of inorganic ions as high as 50000 µg/L. The influence of four organic analogues including phenol, benzoic acid, p-hydroxbenzoic and m-hydroxbenzoic acid at concentrations of 50, 250 and 500 µg/L for each organic analogues respectively on the adsorption of target arsenic species was investigated. As shown in **Table 3**, the allowed concentrations of coexisting analogues were about 500 µg/L or 250 µg/L with a tolerance factor of less than 5%. However, p-NPAA was an exception. The tolerance concentration of p-hydroxybenzoic acid was only 50 µg/L. Possible explanation is shown in **Fig. 7**. Despite other interactions, the hydrogen bonding between hydroxyl on the surface of CM-α-CD-Fe_3_O_4_ and amino groups of p-APAA and AAPAA was very strong but there was hardly any hydrogen bonding between the hydroxyl and nitro of p-NPAA. In any case, phenylarsonic acid could form inclusion complex with α-CD and therefore the arsenate, hydroxyl or amino group were forced closer to the magnetic particles, leading to stronger interactions between the modified material and phenylarsonic acids. Both selectivity and adsorption efficiency were improved compared to that of Fe_3_O_4_ nanoparticles without modification.

**Table 3:** Concentration (µg/L) of coexisting analogues when tolerance factor less than 5%.

	phenol	benzoic acid	p-hydroxbenzoic acid	m-hydroxbenzoic acid
AAPAA	500	500	500	500
p-HPAA	500	250	250	250
p-NPAA	50	500	50	250
p-APAA	>500	500	500	250
ROX	250	500	500	250

### Analytical performance

#### Optimization of UPLC-MS-MS chromatograph separation condition

**Figure 8:**
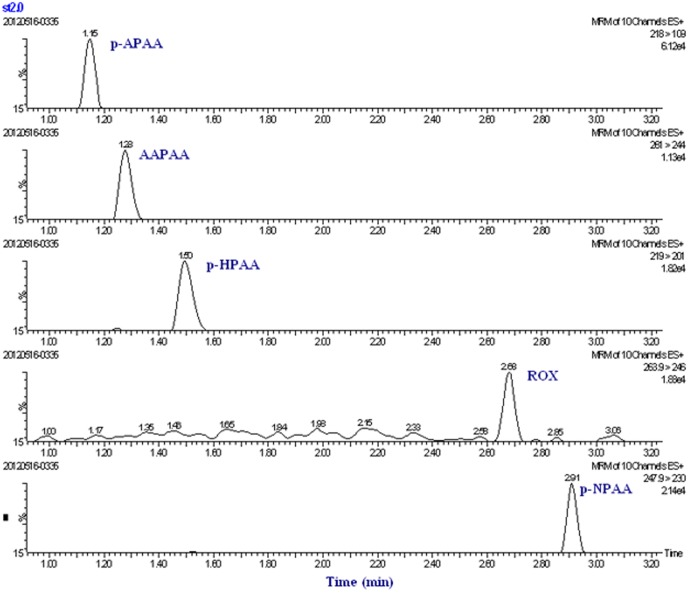
MRM chromatogram of five arsenical compounds standards at 2.0 µg/L.

A mixture of five arsenic compound standards, each at a concentration of 2.0 µg/L, was successfully separated and analyzed by UPLC-MS/MS in less than 3 min on a C18 column. Under the optimal separation conditions, baseline separation was achieved for every arsenic compound. The multi-reaction monitoring (MRM) chromatograms of arsenic compounds are shown in **Fig. 8**.

#### Optimization of sample pretreatment condition

To extract phenylarsonic acids from chicken tissue samples, different extraction methods (including ultrasonic extraction, microwave extraction), extraction time (10, 20, 30, 40, 50 and 60 min) and extraction solvents (methanol, ethanol and toluene) were studied. The optimal recovery was above 75% with the ultrasonic extraction method and toluene as the extraction solvent. The proper extraction time was found to be 30 min and extraction should be conducted twice.

### Method evaluation

The developed method was validated by determining the linearity and LOD of arsenic species listed in **Table 4**. A linear response can be seen in the concentration range of 0.20–10 µg/L (enrichment factor 2.5), with the R^2^ ranging from 0.9951 to 1.0000. The repeatability study was performed for each of the phenylarsonic acids under the optimal conditions. The LOD of each phenylarsonic acid was estimated by analyzing blank samples spiked at 0.2 µg/kg of each target analytes and they were determined as the lowest concentrations of the analyte for which signal-to-noise ratios were 3 respectively. The resultant repeatabilities expressed as RSD varied from 0.85% to 4.49%. These results show that the method has a high sensitivity and good repeatability.

**Table 4:** Analytical performance data with UPLC-MS/MS.

Compounds	Linear range (µg/L)	Linear equation	R^2^	LOD (µg/kg)
p-APAA	0.2–10.0	y = 7305.3x+2211.7	0.9952	0.05
AAPAA	0.2–10.0	y = 2287.2x+398.63	1.0000	0.05
p-HPAA	0.2–10.0	y = 5739.4x+935.79	0.9984	0.10
ROX	0.2–10.0	y = 3249.2x-144.89	1.0000	0.10
p-NPAA	0.2–10.0	y = 1712.3x-87.287	0.9988	0.11

x: the concentration of five phenylarsonic acids, y: peak area. LOD = 3 S/N of blank chicken tissues sample.

### Sample analysis

The phenylarsonic acid in tissue was stored with the prototype compound. So the sample preparation of real tissue samples is same as the spiked samples [17]. In order to validate the suitability of the developed method, the method was applied to analyze spiked chicken tissue samples. For comparison, the total arsenic in these samples was also determined.

**Figure 9:**
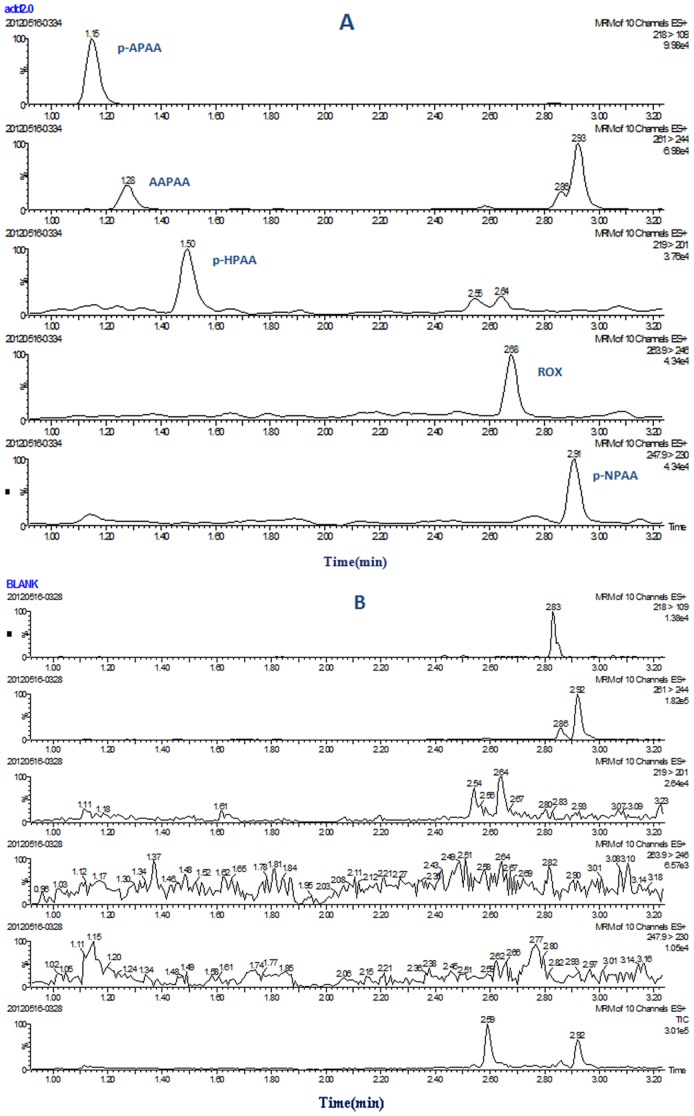
MRM chromatogram of spiked chicken meat (2.0 µg/kg) (A) and blank (B).

In the samples of chicken meat and liver, 0.2, 2 and 20 µg/kg of each phenylarsonic acids were spiked, respectively. The recoveries of five phenylarsonic acids in chicken tissue samples, as shown in **Table 5**, fell in the ranges of 77.2%–110.2%, with a RSD less than 12.5%. The chromatograms of spiked chicken meat sample (2.0 µg/kg) and blank chicken meat sample are shown in **Fig. 9**. To confirm the selectivity of CM-α-CD-Fe_3_O_4_, five phenylarsonic acids were spiked individually in each sample at 0.2 µg/kg and determined by ICP-MS. Recoveries were in the range of 81.5%–119.2% which agreed with the UPLC-MS/MS results. This shows that the CM-α-CD-Fe_3_O_4_ nanoparticles have high selectivity for phenylarsonic acid and can be used for sample clean-up. Combining CM-α-CD-Fe_3_O_4_ nanoparticles clean-up procedure with UPLC-MS-MS technique would potentially result in practical application in the analysis of trace phenyl arsenic acids.

**Table 5:** Recoveries of spiked arsenics in chicken liver and muscle sample (n = 5).

Compounds	Chicken liver			Chicken meat		
	Added (µg/kg)	Recovery (%)	RSD (%)	Added (µg/kg)	Recovery (%)	RSD (%)
p-APAA	0.2	80.7	5.0	0.2	87.3	8.0
	2.0	89.5	6.7	2.0	86.1	7.6
	20	87.7	4.2	20	101.8	5.2
p-HPAA	0.2	94.3	4.9	0.2	77.2	9.1
	2.0	97.9	6.6	2.0	95.1	5.1
	20	93.7	12.5	20	79.9	5.2
p-NPAA	0.2	102.2	4.7	0.2	91.8	7.6
	2.0	110.2	5.4	2.0	98.8	5.7
	20	99.0	10.8	20	102.8	6.3
AAPAA	0.2	82.4	4.4	0.2	83.7	12.1
	2.0	82.6	5.4	2.0	82.6	8.1
	20	78.3	2.0	20	79.6	8.5
ROX	0.2	93.4	2.9	0.2	96.9	10.1
	2.0	104.2	7.3	2.0	104.2	6.4
	20	106.1	4.8	20	108.7	5.2

## Conclusions

In this work, CM-α-CD-Fe_3_O_4_ nanoparticles were synthesized to selectively extract and enrich phenylarsonic acids. CM-α-CD- Fe_3_O_4_ nanoparticles exhibited excellent selectivity and adsorption efficiency for five phenylarsonic acids because of the size selectivity of α-CD and the affinity of Fe_3_O_4_ to arsenic. In the sub ppb level of phenylarsonic acids, the enrichment factor was higher than 400 and the extraction efficiency higher than 70%. Coupled with UPLC-MS/MS, a fast, selective and convenient analytical method for the determination of phenylarsonic acid was developed. Comparing with published method [14], the developed method showed satisfactory sensitivity due to the selectively adsorption of CM-α-CD-Fe_3_O_4_ nanoparticles for phenylarsonic acids in complex sample matrix.

## Supporting Information

Figure S1 **FTIR spectra of Fe_3_O_4_ magnetic nanoparticles (a), CM-α-CD (b) and CM-α- CD- Fe_3_O_4_ (c).**
(TIF)
