# Supplementary figures and images for: Selectively Adsorptive Extraction of Phenylarsonic Acids in Chicken Tissue by Carboxymethyl α-Cyclodextrin Immobilized Fe3O4 Magnetic Nanoparticles Followed Ultra Performance Liquid Chromatography Coupled Tandem Mass Spectrometry Detection
Source: PLoS One. 2014 Sep 12;9(9):e107147. doi: 10.1371/journal.pone.0107147 (PMC4162596; doi:10.1371/journal.pone.0107147)

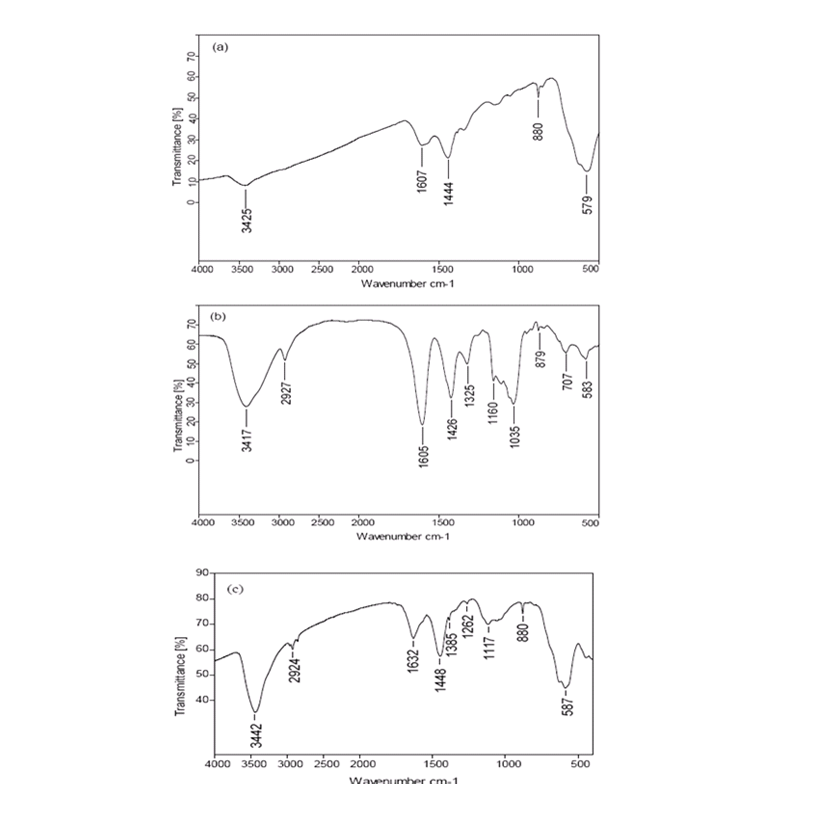

Supplement: Figure S1 — FTIR spectra of Fe3O4 magnetic nanoparticles (a), CM-α-CD (b) and CM-α- CD- Fe3O4 (c). (TIF) [file pone.0107147.s001.tif]
